# The Use of Laser Guidance Reduces Fluoroscopy Time for C-Arm Cone-Beam Computed Tomography-Guided Biopsies

**DOI:** 10.1007/s00270-016-1345-y

**Published:** 2016-04-19

**Authors:** Maarten W. Kroes, Marco J. L. van Strijen, Sicco J. Braak, Yvonne L. Hoogeveen, Frank de Lange, Leo J. Schultze Kool

**Affiliations:** 1 Department of Radiology and Nuclear Medicine, Radboud University Medical Center, P.O. Box 9101, 6500 HB Nijmegen, The Netherlands; 2 Department of Radiology, St. Antonius Hospital, P.O. Box 2500, 3430 EM Nieuwegein, The Netherlands

**Keywords:** C-arm, Cone-beam CT-guidance, Percutaneous biopsy, Laser guidance, Fluoroscopy time

## Abstract

**Purpose:**

When using laser guidance for cone-beam computed tomography (CBCT)-guided needle interventions, planned needle paths are visualized to the operator without the need to switch between entry- and progress-view during needle placement. The current study assesses the effect of laser guidance during CBCT-guided biopsies on fluoroscopy and procedure times.

**Materials and Methods:**

Prospective data from 15 CBCT-guided biopsies of 8–65 mm thoracic and abdominal lesions assisted by a ceiling-mounted laser guidance technique were compared to retrospective data of 36 performed CBCT-guided biopsies of lesions >20 mm using the freehand technique. Fluoroscopy time, procedure time, and number of CBCT-scans were recorded. All data are presented as median (ranges).

**Results:**

For biopsies using the freehand technique, more fluoroscopy time was necessary to guide the needle onto the target, 165 s (83–333 s) compared to 87 s (44–190 s) for laser guidance (*p* < 0.001). Procedure times were shorter for freehand-guided biopsies, 24 min versus 30 min for laser guidance (*p* < 0.001).

**Conclusion:**

The use of laser guidance during CBCT-guided biopsies significantly reduces fluoroscopy time.

## Introduction

Transferring percutaneous needle interventions from a conventional computed tomography (CT)-suite to an interventional suite that uses C-arm cone-beam computed tomography (CBCT) as image guidance technique has several advantages. Firstly, removing the rather time-consuming interventional procedures from the conventional CT-suite increases the patient throughput for diagnostic CT scans [1]. Secondly, the use of CBCT guidance has been reported to improve patient access due to absence of a gantry [2]. Thirdly, CBCT offers the advantages of availability of planning software for double oblique projections and the capability to combine CBCT with a stereotactic navigation device giving the operator the possibility of planning the most optimal needle path from skin entry to target [3, 4]. Lastly, CBCT has been reported to reduce radiation exposure to the patient compared to conventional CT-guided needle interventions [5].

Real-time feedback on the needle position during CBCT-guided needle interventions is provided by fluoroscopy. An earlier phantom study showed that during CBCT-guided needle interventions manipulations of the needle are frequently accompanied by placement of the operator’s hand inside the primary radiation beam [6]. The latter should be avoided whenever possible since hand dose levels can be up to several millisieverts per procedure [7, 8]. By adding laser guidance to the CBCT guidance, radiation exposure was shown to be reduced. The more efficient placement of the needle and fewer corrective needle manipulations minimized direct exposure of the hands to the primary beam and left scatter radiation as the predominant contribution to the hand dose [6].

This study assesses the effect of laser guidance during CBCT-guided biopsies on fluoroscopy and procedure times.

## Materials and Methods

### CBCT Guidance

The CBCT-system in our department is the Allura Xper FD-20 angiosystem (Philips Medical Systems, Best, The Netherlands). In the acquired CBCT-volume, a target is defined and a needle path is planned by the operator. Thereafter, the C-arm is used to guide the needle in real-time along the planned needle path onto the target using fluoroscopy. Two C-arm geometry positions are mainly used to guide a needle: the entry point view, which is an overlay of entry and target point in a bull’s eye fashion, and the progress view, which is perpendicular to entry point view [9].

### Laser Guidance System

**Fig. 1:**
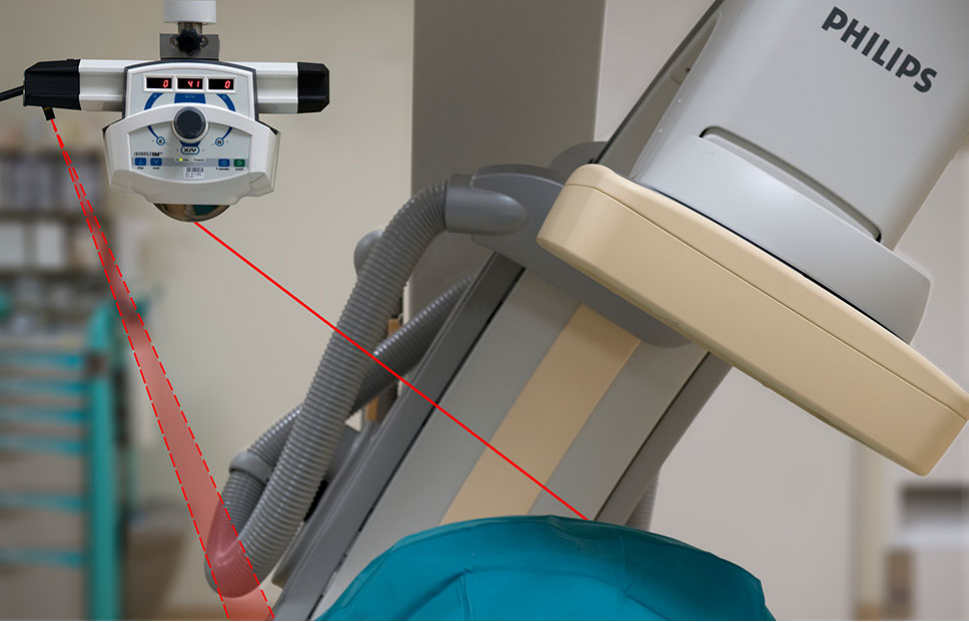
Schematic presentation of the laser guidance setup. The guiding laser of SimpliCT (NeoRad AS, Oslo, Norway) is aimed along a planned needle path of 41° in the axial direction (*straight line*), while the plane laser (*dotted lines*) is aligned to the operating table. The C-arm is positioned in progress view

SimpliCT (NeoRad AS, Oslo, Norway) is a laser-based guidance device for CT-guided percutaneous interventions. The laser guidance acts as a laser pointing device to visualize the planned needle path (possible to 45° in the transversal and sagittal planes) for the operator. For this study, SimpliCT was integrated into the AngioSuite. The battery-driven laser pointer unit was suspended on a short rail from a MAVIG Portegra2 arm. A plane laser was attached to this rail for perpendicular alignment of the laser unit to the C-arm system, using the operating table for horizontal reference alignment (Fig. 1).

### Procedure

**Fig. 2:**
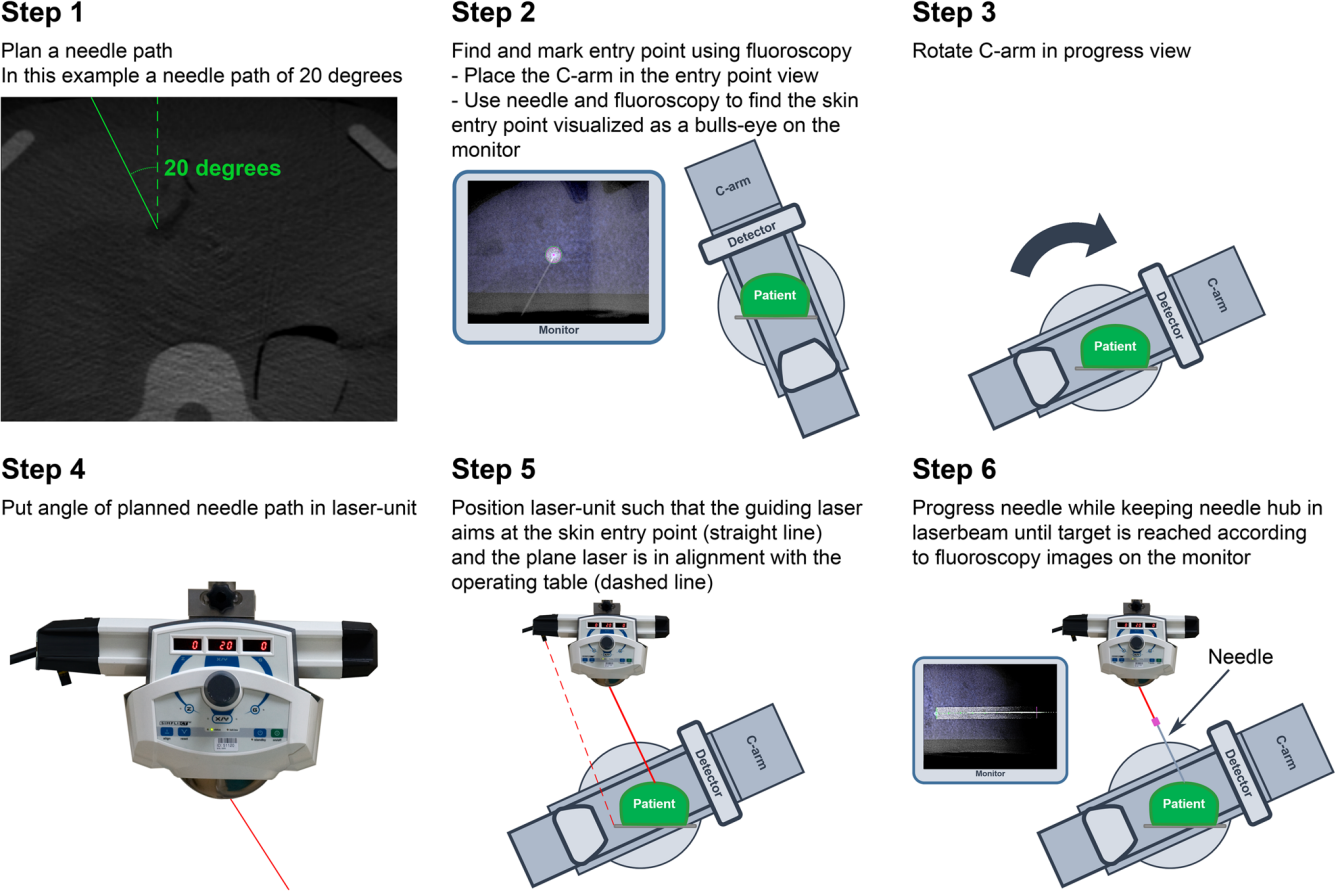
A detailed visualization of the steps during laser guidance in CBCT-guided biopsies

After acquiring the CBCT-volume of the designated patient area the needle path was planned. The angles for the planned needle path, visible in the planning software (XtraVision; Philips Healthcare, Best, The Netherlands), were fed into the laser guidance system. With the C-arm in entry point view, the skin entry point on the patient was found using fluoroscopy and marked. After the C-arm was positioned in progress view, the laser unit was positioned such that the guiding laser was aimed at the marked skin entry point while the plane laser beam was in alignment with the operating table (Fig. 1). With the pointing laser above the skin entry point, the needle was progressed by keeping the needle hub in the laser beam. Fluoroscopy was used to check the needle depth during advancement. These steps are visualized in Fig. 2.

### Patients

A group of 15 patients with an indication for a CBCT-guided biopsy were prospectively included in this study and underwent a CBCT-guided intervention with laser guidance. There were no restrictions in terms of target location or lesion size. Patients had to be able to lie reasonably still and comply with breath-hold commands. All procedures were performed by one interventional radiologist with 4 years of CBCT-guided needle interventions experience (M.J.L.S). Of the 15 biopsies with laser guidance, 7 were thoracic biopsies and 8 were abdominal biopsies. The prospective acquired data were compared to retrospectively acquired CBCT-guided freehand positioned biopsies. To test the hypothesis that laser guidance also reduces fluoroscopy times in a clinical setting, we chose to assess the minimal gain using laser guidance by selecting only relatively easy procedures from the retrospective freehand biopsies. Selection criteria for these biopsies were set at a target size larger than 20 mm in diameter and a procedure time shorter than 35 min. From the total of 82 biopsies, 36 biopsies (16 thoracic and 20 abdominal) met these criteria. All procedures were performed by two interventional radiologists (M.J.L.S. and S.J.B.).

Patient characteristics of both groups are provided in Table 1. The study was exempted for approval by the institutional review board. The laser system is commercially available and is used in standard practice for biopsy procedures.

**Table 1:** Patient and biopsy characteristics

	Laser guidance	CBCT guidance	*p* value
Number patients	15	36	
Age (year)	65 (48–82)	66 (23–85)	*p* = 0.963
Biopsy region (abdominal/thoracic)	8/7	20/16	
Target size min diameter (mm)	15 (8–60)	35.5 (20–93)	*p* < 0.001
Target size max diameter (mm)	20 (10–65)	43 (22–124)	*p* < 0.001
Fluoroscopy (s)	87 (44–190)	165 (83–333)	*p* < 0.001
Procedure time (min)	30 (20–45)	23.5 (3–35)	*p* < 0.001
No. CBCT’s	2 (2–4)	2 (2–4)	*p* = 1

### Outcome Measures

Fluoroscopy time, procedure time, and the number of CBCT-scans were obtained. Fluoroscopy time was defined as the time in seconds of real-time image guidance necessary for placing the needle onto target. These data were extracted from the angiography system software.

Procedure time was defined as the time from the first CBCT until the last taken biopsy. Technical success was defined as the needle tip positioned directly in front of the target or in the target and along the planned needle path. This was measured using the control CBCT images before a biopsy was taken.

### Statistical Analysis

All statistical analyses were conducted in SPSS (version 20.0.0; SPSS Inc., Chicago, USA). All results are represented as medians with corresponding ranges and analyzed using the Mann–Whitney *U* test. Differences were considered statistically significant for *p* < 0.05.

## Results

Technical success was achieved in 100 % of the procedures for both laser-guided and the freehand technique.

**Fig. 3:**
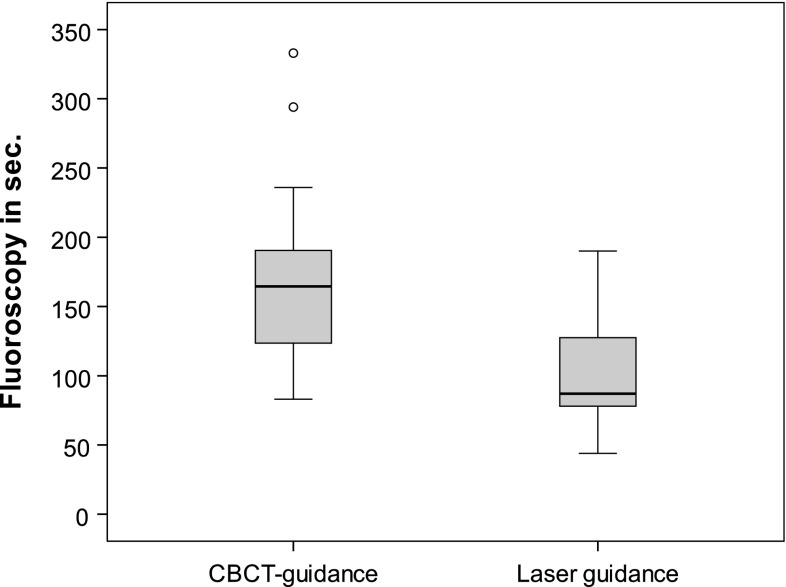
Box plot depicting the fluoroscopy times in seconds required guiding the needle onto the target. Fluoroscopy times for laser-guided biopsies were significantly lower (*p* < 0.001)

In the selected freehand biopsies, median fluoroscopy time required for reaching the target was 165 s (83–333). The median fluoroscopy time for laser-guided biopsies was 87 s (44–190). Comparing these results, the fluoroscopy times were significantly lower (*p* < 0.001) (Fig. 3) in the laser-guided biopsy group.

No significant differences were found in the number of CBCT-scans per procedure. Both techniques used a median of two CBCT-scans (2–4) per procedure.

When comparing procedure times, more time was required for the laser-guided biopsies (30 min) than the selected freehand biopsies (24 min) (*p* = 0.001).

## Discussion

The important finding of this study is that by adding laser guidance to CBCT-guided biopsies there is a significant reduction in fluoroscopy time. Decreasing the fluoroscopy time directly influences the radiation exposure to both the patient and staff. The percentage of fluoroscopy time reduction from freehand to laser guidance is similar to the reduction seen in a laboratory setting using a phantom [6].

The reduction in fluoroscopy time by employing laser guidance is attributable to the visualization of the planned needle path, leading to a more efficient placement of the needle and a reduced number of corrective needle manipulations. Braak et al. [5] and Tselikas et al. [10] found a relatively high contribution of fluoroscopy in the total effective dose to the patient for CBCT-guided needle interventions (35–45 %). In this setting, the clinical operator may therefore also be potentially exposed to high levels of radiation during CBCT-guided needle procedures. Keeping the fluoroscopy time as low as possible should therefore be a target for CBCT-guided procedures.

Alternative strategies aiming to reduce fluoroscopy times have recently been reported. Only a few have been developed specifically for CBCT-guided needle interventions [11–14]. These robotic and electro-magnetic navigational devices can visualize the needle position in the scanned volume in real-time but require additional installment times for each procedure. Recently, Ritter et al. reported the use of a crosshair laser integrated into the detector housing of an angiography system as an aid to reduce fluoroscopy time in CBCT-guided procedures. The crosshair laser was used to visualize the needle entry point on the skin of the patient on the basis of the planned path with the C-arm in entry point view [15, 16]. In progress view, however, with the C-arm perpendicular to the planned needle path, the crosshair laser is unable to help the operator in maintaining the correct angle of the needle during progression. The effect on fluoroscopy time using laser guidance was not assessed in the Ritter study.

In the current study, there were no differences in CBCT-scans between the freehand technique and laser guidance. By giving breathing instructions and instructing the patient not to move during the procedure we were able to minimize the number of CBCT-scans, which is a standard protocol for both techniques.

There are several limitations to the current study. First of all, prospective laser guidance data were compared to retrospective data. All performed CBCT-guided needle interventions were collected in a database since the installation of our CBCT-system. To be able to analyze laser guidance performance in an efficient manner, this study was therefore setup as a retrospective study. Second, to challenge the test toward the hypothesized effect on fluoroscopy time reduction using laser guidance, we selected only the easiest freehand-guided biopsies based on target size and procedure time. The consequential dissimilarity in procedures likely affected the relative effect on fluoroscopy and procedure times reported in this study. To overcome these limitations, a large prospective study is required in the future.

Compared to the freehand technique, the use of laser guidance lengthened the procedure time by 6 min. This difference is probably caused partly by the extra time required for setting up the laser system, and partly by the selected freehand group since this group was selected not only on lesion size >20 mm but also on the shortest procedure times.

## Conclusion

In conclusion, this study indicates that adding laser guidance to CBCT-guided biopsies provides visual feedback that significantly reduces fluoroscopy time, and consequently assists in reducing radiation exposure to both patient and interventional staff. In daily clinical practice, the cost in terms of prolonged procedure times will probably be marginal.
